# A Feasibility Study Assessing a Novel Ambulatory Monitoring System in General Ward Areas: Pilot Study

**DOI:** 10.1002/nop2.70213

**Published:** 2025-04-10

**Authors:** Meera Joshi, Pavidra Sivanandarajah, Michael Wright, John Beard, Sadia Khan

**Affiliations:** ^1^ Department of Surgery Imperial College London UK; ^2^ Department of Cardiology Chelsea and Westminster Hospital NHS Foundation Trust London UK; ^3^ Innovation, Chelsea and Westminster Hospital NHS Foundation Trust London UK; ^4^ GE HealthCare Chicago USA; ^5^ National Heart and Lung Institute Imperial College London UK

## Abstract

**Aim:**

To assess the clinical staff and patient acceptance of a novel ambulatory monitoring system in general medical and surgical inpatient wards.

**Design:**

This was a cross‐sectional feasibility study undertaken in a single hospital site.

**Methods:**

Patients and the nursing staff were asked to provide feedback on a monitoring system via structured questionnaires while enrolled in an observational monitoring system study. Patient recruitment occurred in the general ward areas (both surgical and medical). Patients were asked to wear the ambulatory monitoring system, and physiological data were sampled, whereas staff were asked to train on the monitoring system and utilise it in clinical care.

**Results:**

Questionnaire feedback collected from 27 staff surveyed showed that 96% of responses reported the system was good or very good to use. Thirty‐four participants wore the monitoring system for an average duration of 21 h, and 91% reported it was comfortable to wear.

**Patient or Public Contribution:**

Patients participated in the design of this feasibility study.

## 1 Introduction

Failure to recognise clinical deterioration is catastrophic for patients, health care staff and health care systems (Findlay et al. 2012; Adam and Odell 2005; Herasevich et al. 2022; Peelen et al. 2023). Typically, deterioration is detected by intermittent measurement of physiological parameters at the bedside at intervals separated by hours. Additionally, current devices used for vital sign measurement do not measure respiratory rate (RR), increasingly recognised as an early marker of deterioration. RR is recorded manually with notorious inaccuracy (Cretikos et al. 2008; Latten et al. 2019; Churpek et al. 2018).

Track and trigger surveillance systems, using standardised early warning scores (EWS) (Peelen et al. 2023; Royal College of Physicians 2017) and bedside physiological parameters, have been developed in response to this patient safety challenge. Recording of EWS is time‐consuming and frequently dependent on manual entries by nursing staff, which presents a significant patient safety risk. Despite the availability of these track and trigger systems, deterioration often remains unrecognised (Peelen et al. 2023; NICE Clinical Guidelines 2007).

## 2 Background

These challenges are occurring against the backdrop of significant staffing challenges worldwide, with high vacancy rates and burnout following the pandemic, factors that will invariably amplify the safety challenge. Technology‐based continuous physiologic monitoring solutions may support nurse surveillance of groups of patients and identify those patients requiring higher level monitoring while providing data for EWS. Early identification of patient deterioration has been shown to lead to more effective and timely interventions (Grønbæk et al. 2023) and support nursing staff retention and experience of work through more efficient and effective care models. Conversely, technologies that provide non‐actionable data or create additional workload through excess alerts may exacerbate current challenges and further erode all staff productivity and morale (Ancker et al. 2017). Monitoring platforms that continuously and wirelessly obtain physiologic data are increasingly available to health systems and offer significant opportunities to meet these challenges (Joshi et al. 2022; Bates et al. 2023). However, challenges to the adoption of continuous monitoring systems include patient comfort, staff satisfaction and cost. Against this backdrop, we aimed to evaluate the clinical experience using a novel wearable system on general surgical and medical ward areas with a particular focus on end‐user experiences of using this system, including both nursing staff and patients.

## 3 The Study

### 3.1 Aim and Objective

The aim of this feasibility study was to assess bedside providers' and patients' perception of a novel wearable wireless physiologic monitoring system in medical and surgical ward areas in a general hospital setting. Patients in these areas normally receive intermittent vital signs assessment rather than continuous monitoring.

### 3.2 Primary and Secondary Objective

The primary objective was to assess the clinical staff's perception of the monitoring system. The secondary objective was to assess the patient perceptions and experiences of the monitoring system.

## 4 Methods

### 4.1 Study Design

This feasibility study is a cross‐sectional component of an observational clinical study. The study protocol was developed in accordance with recommendations from the Standard Protocol Items: Recommendations for Interventional Trials guidelines. All participants provided informed consent. Ethics approval was granted by the Stanmore Ethics Committee (IRAS reference 285644). Storage and the handling of personal data complied with the General Data Protection Regulation. The cross‐sectional feasibility study included a survey tool, administered after using the monitoring technology for capturing clinician opinion and overall feedback (Appendix 1). Bedside providers were surveyed after serving as caregivers for a patient who wore the monitoring technology. Patients would have to wear the monitor for at least 30 min before they were asked to provide feedback. All qualified nursing staff who provided care for a patient wearing the monitor for over 30 min could complete the staff tool. The tool was created by the research site in collaboration with the study sponsor and did not include formal psychometrics.

### 4.2 Study Participants and Setting

The clinical study was undertaken at the West Middlesex Univeristy Hospital, London from March 2021 until June 2021. This is a medium‐sized (400 beds) general hospital; data were collected on both general medical and surgical clinical units. After morning patient care rounds were complete, the medical and surgical patient‐care teams provided a daily list of patients estimated to remain in hospital for at least an additional 24 h and who they had assessed as being able to provide their informed consent. Patients aged 18 years and older were eligible for inclusion in the feasibility study. Eligible patients were approached by the research team and given a feasibility study information sheet. Participants were asked to wear the monitoring system for a minimum of 20 min and up to a maximum of 72 h. All patients were recruited from 10 March 2021 to 15 June 2021. Exclusion criteria included the presence of implantable cardiac devices, sensitivity to the monitoring electrodes, a primary admission diagnosis for a mental health condition, allergies to the dressings or tape being used in their care, or anticipated discharge from hospital within the next 24 h. In addition, any patients unable to provide their consent for any reason were not eligible for inclusion in the feasibility study. There were no exclusion criteria for bedside providers to participate in the feasibility study. Clinicians were eligible to complete a survey after caring for one or more patients and had no minimum level or type of required clinical experience.

### 4.3 Ambulatory Monitoring System

**FIGURE 1:**
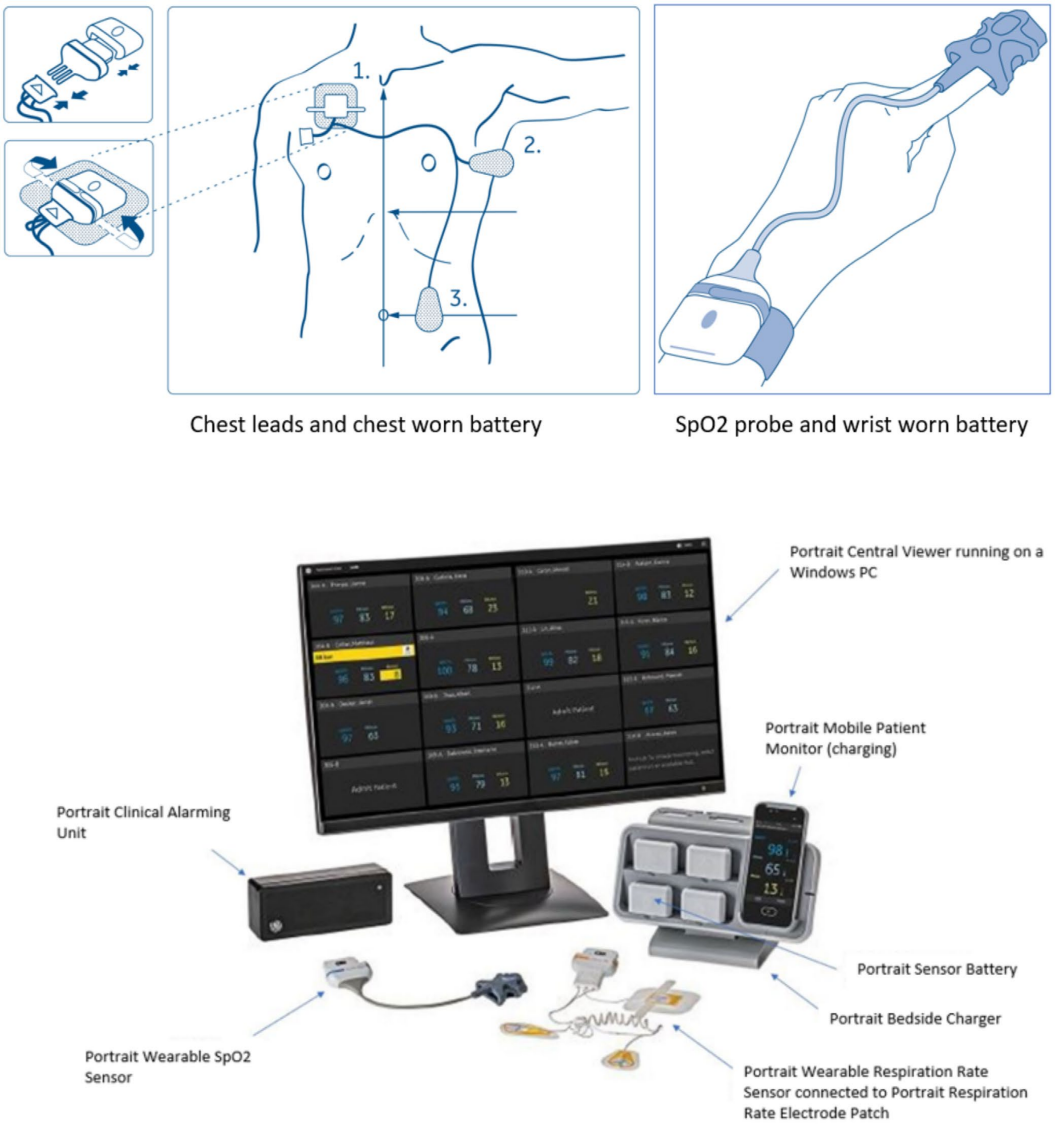
The portrait mobile system showing sensor position and components.

Individuals recruited into the feasibility study were fitted with an ambulatory monitoring system (Portrait Mobile; GE HealthCare, Chicago, IL, USA) by the medical–surgical clinical staff providing clinical care. The ambulatory monitoring system (van Melzen et al. 2023) is a wireless and wearable technology that enables continuous monitoring of ambulatory patients' RR, SpO_2_ and pulse rate (PR) (Figure 1).

The ambulatory monitoring system collects physiologic data using wireless sensors that are worn by the patient, with data transmitted to either or both a bedside console and a central monitoring hub. RR is measured using a chest‐worn, disposable three‐lead system with a rechargeable battery (TruSignal RRdv; GE HealthCare). Oxygen saturation and PR are measured using a fingertip pulse oximetry probe with a wrist‐mounted rechargeable battery (TruSignal SpO_2_; GE HealthCare). During this feasibility study, the vital signs of RR, SpO_2_, PR, blood pressure and temperature were also collected using their standard of care routine measurement, as the clinical staff was blinded to the study device collecting the wireless data.

Recorded monitoring data without patient identifiers was stored on the hospital network and was accessible only by research personnel. Live data were visible in real time to ward staff.

Participants and ward staff were asked to anonymously complete questionnaires about their experience of the system. Clinicians and subjects completed their surveys on paper source forms distributed by study staff and then their responses were transcribed into the INFORM electronic CRF database by the feasibility study staff under the respective clinician or subject IDs. The transcription was verified by additional research staff during monitoring visits. These questionnaires are included in Appendices 1 and 2.

### 4.4 Statistical Analysis

Acceptability and usability of the monitoring system by participants and healthcare staff were evaluated via questionnaires, including 5‐point Likert scale responses (strongly disagree to strongly agree), along with open‐ended questions. Descriptive statistics were used to describe baseline characteristics of patients and bedside providers. Response distribution analysis was conducted for Likert scale questions, whereas subjective responses were collected for internal investigator and sponsor data. There were also some open‐ended questions included. This feasibility study report follows the STROBE guidelines with the checklist included in Table S1.

### 4.5 Power Considerations

This feasibility study was not powered to detect any change in clinical outcomes. However, based on our previous work and sample sizes recommended in US Food and Drug Administration human factor guidance, we calculated that a sample size of 15–20 patients would be needed to assess usability of the system and to gather meaningful feedback for thematic saturation to be achieved (Becking‐Verhaar et al. 2023). As a feasibility study, statistical significance of the findings was not expected.

## 5 Results

The results of this feasibility study are both quantitative and qualitative in nature and serve as a starting point for future scientific inquiry, particularly as it relates to clinical outcomes and cost‐effectiveness. Quantitative data are used for descriptive purposes.

### 5.1 Subjects

Thirty‐four patients were recruited with the baseline characteristics as per Table 1. One participant withdrew from the feasibility study, and only the baseline data are presented here. Twenty‐seven clinicians participated in the feasibility study with characteristics listed in Table 2.

**TABLE 1:** Baseline characteristics of patient participants.

Total number	34
*Gender*	
Male	23
Female	11
Mean age (range)	58.4 years (26–81 years)
*Ward bed base*	
Medical	18
Surgical	16
Mean weight (range)	82 kg (52–145 kg)
Nursed in bed	3
Primarily in bed	7
Ambulant in room	4
Fully ambulant^a^	20

^a^ Fully ambulant patients were freely mobile throughout the ward area.

**TABLE 2:** Baseline characteristics of clinician participants.

Total number	27
*Clinician type*	
Band 5 nursing staff	20
Band 6 or above nursing staff	5
Registered nurse	1
Research clinical fellow	1
Mean length of experience (range)	6 years (1–21 years)
*Years of experience working with investigational technologies*	
< 1 year	8
1–5 years	13
6–10 years	6

### 5.2 Monitoring Data

On the 34 patients included in the feasibility study, over 38,100 min of data from monitoring was obtained with over 2 million data points. Per patient average duration of monitoring was 21 h (range: 1.9–75.4 h).

### 5.3 Patient and Staff Feedback

Feedback from the 27 staff members is summarised in Table 3. Participants were asked to give an overall rating to the use of the monitoring system, with 92% rating the system as good or very good to use. Feedback was collected from 34 patient participants in the feasibility study, with 91% agreeing that the sensors were comfortable or very comfortable to wear, and 76% agreeing or strongly agreeing that they felt safer by wearing the system. The questionnaires used are included in Appendices 1 (staff feedback) and 2 (patient feedback).

**TABLE 3:** Feedback from staff.^a^

	Strongly agree *N* (%)	Agree *N* (%)	Neither agree nor disagree *N* (%)	Disagree *N* (%)	Strongly disagree *N* (%)
I feel more reassured about my patient's condition when continuous monitoring is used vs. vital signs spot check measuring	7 (26)	17 (63)	3 (11)	0	0
I am more confident in assessing respiratory function using this remote monitoring	6 (22)	14 (52)	7 (26)	0	0
This patient monitoring system increases patient safety	8 (30)	11 (41)	8 (30)	0	0
This patient monitoring system could help in earlier detection of patient deterioration than routine observations	6 (22)	20 (74)	1 (4)	0	0
This patient monitoring system supports earlier patient mobilisation	2 (7)	12 (44)	10 (37)	3 (11)	0

*Note:* Table summarised data collected from 27 clinicians. Clinicians may have submitted a response for each individual enrolled patient they cared for, thus submitting more than one. The responses in this analysis represent the final response of an individual clinician in the study which may be most representative of their experience with the system.

^a^ Data presented as *N* (%).

In addition to assessing overall usability, clinicians were surveyed on various aspects of clinical workflow and perceptions of patient's impact. These additional results are presented in Table 3.

## 6 Discussion

The ongoing need for improvements in patient safety has been reinforced by recognising that adverse events during hospitalisation continue to occur and that many of these are preventable (Bates et al. 2023). Prevention of clinical deterioration may be driven through newly developed wearable monitoring systems that continuously measure patient physiologic data and can alert care givers to changes in patient condition (van Melzen et al. 2023; Joshi et al. 2021). These new monitoring systems cannot be presumed to address all safety issues, as the introduction of any new technology into a hospital is a complex endeavour with multiple compounding factors, including patient characteristics, nurse engagement and alarm burden (van Melzen et al. 2023; Becking‐Verhaar et al. 2023; Bitkina et al. 2020).

This feasibility study describes the use of a novel wireless wearable monitoring system in the acute care setting on the medical–surgical clinical units and the experiences of patients and clinicians. The patients had a limited role in the workflow associated with the technology; thus, the outcomes associated with participation were focused on outcomes related to psychological and physical comfort. The results suggest that continuous monitoring can provide patients with a sense of reassurance of safety, perhaps arising from a perception that they were being looked after even when providers were not present. It was also reported that the device was physically comfortable, which is essential for monitoring to be successful over time. A technology that causes physical discomfort may limit activity, interfere with sleep and lead patients and or nurses to opt out of the monitoring system. Patient reassurance may be an important driver in the development of healthcare consumer demand, which could lead to expectations on the healthcare system to provide continuous monitoring technology routinely. Health systems that provide continuous monitoring should be able to provide better and safer care and may have a perceived advantage in the marketplace when competing for patients, driving further technology adoption and implementation.

Also critically important is the staff experience with the technology (Becking‐Verhaar et al. 2023). These results suggest that the monitoring system was viewed favourably as staff indicated that use of the system was good or very good in 96% of responses. Consistent with previous research, a positive clinician experience at the point of care should contribute to better acceptance of new technologies (Bitkina et al. 2020). Most clinicians reported increased reassurance of patient safety and perception of improved respiratory monitoring. A sense of safety driven by continuous monitoring may contribute to improved perception of the effectiveness of care and increased provider professional satisfaction.

The survey question where there was disagreement among respondents was assessing early mobilisation, where 44% felt the system favoured early mobilisation, 43% were neutral and 12% disagreed. The system is wireless and wearable and comes with a recommendation to ambulate outside the room along with the systems monitoring hub. Although not clear from the responses, either the body worn monitor or carrying the hub could contribute to a perception that early mobilisation is not facilitated. On the contrary, continuous vital sign monitoring during ambulation may provide reassurance of physiologic stability, providing confidence to mobilise more actively during recovery.

Technical performance of the monitoring system is an important element for adoption and compliance as the ratio of actionable to non‐actionable alerts may contribute to clinical utility or conversely alarm fatigue. In this feasibility study, the technical performance of the monitor was not the primary focus although consistent physical patient contact points, wireless connectivity and battery life are important for these systems to deliver high levels of connectivity and compatibility with clinical workflows. As with patient and clinician experience, technical performance is important for monitoring system adoption and compliance and will be the subject of future investigation (Leenen et al. 2023).

### 6.1 Strengths and Limitations of the Work

This feasibility study was undertaken in general medical–surgical units during the COVID‐19 pandemic. This is likely to be both a strength and a limitation in that the pandemic response drove the adoption of continuous monitoring solutions to reduce providers' patient infectious exposure. A significant limitation is the lack of technical and clinical outcome measures linked to this system, and this should be addressed by further research.

### 6.2 Recommendations for Further Research

Further research should include study of clinical outcomes, detection of deterioration and impact of workflows and alarm fatigue on nursing staff. Technology evaluation should include study of battery life, wireless connectivity and the incidence of technical versus clinical alarms during care. Research may address whether health systems investing in continuous monitoring technology develop a competitive advantage in recruiting and retaining staff. Perceptions of effectiveness and satisfaction among caregivers could be assessed in relation to clinical results and avoidance of traumatic episodes of patient harm. Efficiency investigations may include the impact of automated documentation of vital signs on workflows and timely health record entries and early warning score updates.

## 7 Conclusion

Wireless wearable physiologic monitoring systems have the potential to improve in‐hospital care by supporting earlier detection of patient deterioration and facilitating effective interventions. Positive patient and caregiver experience is essential for the implementation, adoption and high compliance needed for wearable technologies to provide the continuous clinical data streams required to effectively monitor clinical conditions and provide data for EWS. This study presents early evidence that a wireless, wearable system may meet patient and caregiver needs with the potential for effective future deployment.

## Author Contributions

Meera Joshi: conceptualisation, methodology, investigation, analysis, funding acquisition, writing. Pavidra Sivanandarajah: investigation, analysis, writing. Michael Wright: conceptualisation, methodology, investigation, analysis, funding acquisition, project administration, writing. John Beard: formal analysis, writing. Sadia Khan: conceptualisation, methodology, investigation, analysis, funding acquisition, writing, project administration, supervision.

## Conflicts of Interest

S.K. and M.J. have undertaken consulting for GE HealthCare. J.B. is employed by GE HealthCare. M.W. and P.S. have no conflicts to declare.

## Supporting information


Table S1.

## Data Availability

The data that support the findings of this study are available from the corresponding author upon reasonable request.
