# Correction to ‘A Feasibility Study Assessing a Novel Ambulatory Monitoring System in General Ward Areas: Pilot Study’

**DOI:** 10.1002/nop2.70390

**Published:** 2025-12-23

Joshi, M., P. Sivanandarajah, M. Wright, J. Beard, S. Khan. 2025. “A Feasibility Study Assessing a Novel Ambulatory Monitoring System in General Ward Areas: Pilot Study.” *Nursing Open* 12, no. 4: e70213. https://doi.org/10.1002/nop2.70213.

In section ‘5.3 Patient and Staff Feedback’ the text ‘Feedback from the 27 staff members is summarised in Table 3. Participants were asked to give an overall rating to the use of the monitoring system, with 92% rating the system as good or very good to use’ was incorrect. This should have read ‘Feedback from the 27 staff members is summarised in Table 3. Participants were asked to give an overall rating to the use of the monitoring system, with 96% rating the system as good or very good to use’.

We apologise for this error.

