# Supplementary material for: A Feasibility Study Assessing a Novel Ambulatory Monitoring System in General Ward Areas: Pilot Study
Source: Nurs Open. 2025 Apr 10;12(4):e70213. doi: 10.1002/nop2.70213 (PMC11985360; doi:10.1002/nop2.70213)
Supplement: Supplementary file 1 — Table S1. [file NOP2-12-e70213-s001.docx]

**Supplementary Table 1**

STROBE Statement—checklist of items that should be included in reports of observational studies

|  | | | | **Item No.** | | **Recommendation** | | | **Page  No.** | | |  |  |
| --- | --- | --- | --- | --- | --- | --- | --- | --- | --- | --- | --- | --- | --- |
| **Title and abstract** | | | | 1 | | (*a*) Indicate the study’s design with a commonly used term in the title or the abstract | | | 1 | | |  |  |
|  |  |  |  |  |  | (*b*) Provide in the abstract an informative and balanced summary of what was done and what was found | | | 1 | | |  |  |
| **Introduction** | | | | | | | | | | | |  |  |
| Background/rationale | | | | 2 | | Explain the scientific background and rationale for the investigation being reported | | | 2 | | |  |  |
| Objectives | | | | 3 | | State specific objectives, including any prespecified hypotheses | | | 2 | | |  |  |
| **Methods** | | | | | | | | | | | |  |  |
| Study design | | | | 4 | | Present key elements of study design early in the paper | | | 2 | | |  |  |
| Setting | | | | 5 | | Describe the setting, locations, and relevant dates, including periods of recruitment, exposure, follow-up, and data collection | | | 3 | | |  |  |
| Participants | | | | 6 | | (*a*) *Cohort study*—Give the eligibility criteria, and the sources and methods of selection of participants. Describe methods of follow-up  *Case-control study*—Give the eligibility criteria, and the sources and methods of case ascertainment and control selection. Give the rationale for the choice of cases and controls  *Cross-sectional study*—Give the eligibility criteria, and the sources and methods of selection of participants | | | 3 | | |  |  |
|  |  |  |  |  |  | (*b*) *Cohort study*—For matched studies, give matching criteria and number of exposed and unexposed  *Case-control study*—For matched studies, give matching criteria and the number of controls per case | | |  | | |  |  |
| Variables | | | | 7 | | Clearly define all outcomes, exposures, predictors, potential confounders, and effect modifiers. Give diagnostic criteria, if applicable | | | 3 | | |  |  |
| Data sources/ measurement | | | | 8* | | For each variable of interest, give sources of data and details of methods of assessment (measurement). Describe comparability of assessment methods if there is more than one group | | | 3 | | |  |  |
| Bias | | | | 9 | | Describe any efforts to address potential sources of bias | | | N/a | | |  |  |
| Study size | | | | 10 | | Explain how the study size was arrived at | | | 4 | | |  |  |
| Continued on next page Quantitative variables | | 11 | | | Explain how quantitative variables were handled in the analyses. If applicable, describe which groupings were chosen and why | | | 4 | | |  | | |
| Statistical methods | | 12 | | | (*a*) Describe all statistical methods, including those used to control for confounding | | | 4 | | |  | | |
|  |  |  |  |  | (*b*) Describe any methods used to examine subgroups and interactions | | | N/A | | |  | | |
|  |  |  |  |  | (*c*) Explain how missing data were addressed | | | N/A | | |  | | |
|  |  |  |  |  | (*d*) *Cohort study*—If applicable, explain how loss to follow-up was addressed  *Case-control study*—If applicable, explain how matching of cases and controls was addressed  *Cross-sectional study*—If applicable, describe analytical methods taking account of sampling strategy | | | N/A | | |  | | |
|  |  |  |  |  | (*e*) Describe any sensitivity analyses | | | N/A | | |  | | |
| **Results** | | | | | | | | | | | | | |
| Participants | | 13* | | | (a) Report numbers of individuals at each stage of study—eg numbers potentially eligible, examined for eligibility, confirmed eligible, included in the study, completing follow-up, and analysed | | | 5 | | |  | | |
|  |  |  |  |  | (b) Give reasons for non-participation at each stage | | | N/A | | |  | | |
|  |  |  |  |  | (c) Consider use of a flow diagram | | | N/A | | |  | | |
| Descriptive data | | 14* | | | (a) Give characteristics of study participants (eg demographic, clinical, social) and information on exposures and potential confounders | | |  | | |  | | |
|  |  |  |  |  | (b) Indicate number of participants with missing data for each variable of interest | | | 5 | | |  | | |
|  |  |  |  |  | (c) *Cohort study*—Summarise follow-up time (eg, average and total amount) | | | 5 | | |  | | |
| Outcome data | | 15* | | | *Cohort study*—Report numbers of outcome events or summary measures over time | | | 6 | | |  | | |
|  |  |  |  |  | *Case-control study—*Report numbers in each exposure category, or summary measures of exposure | | |  | | |  | | |
|  |  |  |  |  | *Cross-sectional study—*Report numbers of outcome events or summary measures | | |  | | |  | | |
| Main results | | 16 | | | (*a*) Give unadjusted estimates and, if applicable, confounder-adjusted estimates and their precision (eg, 95% confidence interval). Make clear which confounders were adjusted for and why they were included | | | 6 | | |  | | |
|  |  |  |  |  | (*b*) Report category boundaries when continuous variables were categorized | | | N/A | | |  | | |
|  |  |  |  |  | (*c*) If relevant, consider translating estimates of relative risk into absolute risk for a meaningful time period | | | N/A | | |  | | |
| Continued on next page Other analyses | 17 | | Report other analyses done—eg analyses of subgroups and interactions, and sensitivity analyses | | | | 6 | | |  | | | |
| **Discussion** | | | | | | | | | | | | | |
| Key results | 18 | | Summarise key results with reference to study objectives | | | | 7 | | |  | | | |
| Limitations | 19 | | Discuss limitations of the study, taking into account sources of potential bias or imprecision. Discuss both direction and magnitude of any potential bias | | | | 7 | | |  | | | |
| Interpretation | 20 | | Give a cautious overall interpretation of results considering objectives, limitations, multiplicity of analyses, results from similar studies, and other relevant evidence | | | | 7 | | |  | | | |
| Generalisability | 21 | | Discuss the generalisability (external validity) of the study results | | | | 8 | | |  | | | |
| **Other information** | | |  | | | | | | | | | | |
| Funding | 22 | | Give the source of funding and the role of the funders for the present study and, if applicable, for the original study on which the present article is based | | | | 8 | | |  | | | |

*Give information separately for cases and controls in case-control studies and, if applicable, for exposed and unexposed groups in cohort and cross-sectional studies.
